# Relevantes interprofessionelles Wissen des (Allgemein‑/Viszeral‑)Chirurgen – Informationssicherheit und Datenschutz im chirurgischen Klinikalltag

**DOI:** 10.1007/s00104-022-01764-y

**Published:** 2022-11-28

**Authors:** André Helm, Jana Fruth, Ute Klanten, Susan Rönnebeck, Frank Meyer

**Affiliations:** Klinik für Allgemein‑, Viszeral‑, Gefäß- und Transplantationschirurgie, Universitätsklinikum Magdeburg A.ö.R., Leipziger Straße 44, 39120 Magdeburg, Deutschland

**Keywords:** IT-Notfallmanagement, Patientensicherheit, Behandlungseffektivität, Klinischer Alltag, Informationssicherheitsmanagementsystem, IT emergency management, Patient safety, Treatment effectiveness, Daily clinical practice, Information safety management system

## Abstract

**Ziel und Methode:**

Kurzübersicht, basierend auf gesetzlichen Vorgaben, individuellen Erfahrungen aus der täglichen Praxis und ausgewählten Referenzen der aktuellen Literatur.

**Ergebnisse (selektive Eckpunkte):**

Informationssicherheit und Datenschutz sind gesetzlich vorgeschriebene und unverzichtbare Elemente allgemeinen, beruflichen und sozialen Handelns, die auch ins Qualitätsmanagement (QM) eingehen, dem auch der (Allgemein-/Viszeral-)Chirurg in seinem Wirken untersteht.

**Informationssicherheit:**

Sie bezeichnet den Schutz von Informationen jeglicher Art und Herkunft - sie dient der Sicherstellung der Grundziele Vertraulichkeit, Integrität, Verfügbarkeit und Authentizität von Informationen, die die beiden Ziele des Klinikers, Patientensicherheit und Behandlungseffektivität, unterstützt.

**Datenschutz:**

Es handelt sich dabei um ein Grundrecht zum Schutz vor missbräuchlicher Verarbeitung personenbezogener Daten - dabei ist nicht der Schutz der Daten, sondern das Recht des Einzelnen auf informationelle Selbstbestimmung das Schutzziel.

**IT-Notfallmanagement:**

Es umfasst, dass man im IT-Notfall (ausgelöst z. B. durch Systemausfall oder Cyberangriff) geordnet und unterbrechungsfrei in einen vorher definierten klinischen Notfallbetrieb wechselt - aus digitalen werden in ausschließlicher Instituts- bzw. Klinikverantwortung befindliche analoge Prozesse.

**Schlussfolgerung:**

Von einem regelmäßigen bilateralen Austausch zwischen dem (Allgemein-/Viszeral-)Chirurgen und den Beauftragten für Informationssicherheit und Datenschutz profitieren beide Seiten gleichermaßen. Sie sind dadurch in der Lage, die gemeinsamen Ziele der Patientensicherheit und der Behandlungseffektivität durch eine verhältnismäßige Integration von Informationssicherheit und Datenschutz in den klinischen Alltag sicher zu stellen.

## Abstract (en)

**Aim and Method:**

Short narrative overview based on regulations by law, individual experiences from daily practice and selected references from current literature.

**Results (selective corner points):**

Information security and data protection are mandatory and indispensable elements by law of general, professional and social actions, which can be also considered substantial parts of quality management. Therefore, (general/abdominal) surgeon underlies it too.

**Information security:**

It comprises the protection of information of each type and origin - it serves the warranty of the basic aims such as confidentiality, integrity, availability and authenticity of information, which supports both aims of a clinician, patient safety and treatment effectiveness.

**Data protection:**

It is considered a basic right to protect personal data against abusive processing - however, data security is not the primary aim, but rather the right for informational self-determination.

**IT emergency management:**

It implies that in case of an IT emergency (released by a persisting system failure or cyberattack), logistic operations are transferred into previously defined emergency procedures well organized and possibly with no interruption - digital logistic operations become suddenly complete analogous processes, with exclusive responsibility of the single institutes and departments.

**Conclusion:**

Both sides benefit equally from a regular bilateral exchange between the surgeon and the information security and data protection officer and are able to ensure the common goals of patient safety and treatment effectiveness through a proportionate integration of information security and data protection into everyday clinical practice.

## Einleitung

Die digitale Entwicklung führt zu einer immer umfangreicheren und schnelleren sowie vernetzten Verarbeitung von Daten im Klinikalltag.

Dabei sind *Informationssicherheit* und *Datenschutz* heutige Kernelemente einer adäquaten modernen Arbeitsorganisation und ihrer stetigen Optimierungsbemühungen – ihre Rolle für den geschäftlichen Erfolg eines Unternehmens ist nicht zu unterschätzen.

Um hierbei einen ausreichenden Schutz sicherzustellen, wurden im Bereich der Informationssicherheit und des Datenschutzes in den letzten Jahren zahlreiche neue Regelungen eingeführt [1].

Mit der Einführung des IT(Informationstechnologie)-Sicherheitsgesetzes im Jahr 2017 hat der Gesetzgeber sog. „kritische Infrastrukturen“ identifiziert [2, 3]. Im Sektor Gesundheit sind das alle Kliniken und Krankenhäuser mit mindestens 30.000 vollstationären Fällen pro Jahr [4].

Diese kritischen Infrastrukturen sind laut Gesetz verpflichtet, ein „Informationssicherheitsmanagementsystem“ (kurz: ISMS) einzuführen und auf dessen Grundlage Anforderungen der Informationssicherheit im Klinikbetrieb umzusetzen. Ein Nachweis dafür muss alle zwei Jahre gegenüber dem „Bundesamt für Sicherheit in der Informationstechnologie“ (kurz: BSI) im Rahmen extern durchgeführter Audits erbracht werden. Zur Erfüllung dieser Ziele ist auch ein IT-Notfallmanagement, nicht zuletzt als wesentlicher Bestandteil eines übergeordneten betrieblichen Kontinuitätsmanagements („business continuity management“, kurz: BCM), unverzichtbar [5–8].

Der Datenschutz ist in Deutschland mit dem Schutz des Rechts auf informationelle Selbstbestimmung bereits im Grundgesetz verankert. Weiterhin gilt seit dem 25.05.2018 die EU-Datenschutz-Grundverordnung (DSGVO; [17]), in der die Verarbeitung personenbezogener Daten europaweit geregelt ist. Neben der DSGVO gibt es mit dem Bundesdatenschutzgesetz [18] und den Datenschutzgesetzen der einzelnen Bundesländer [19] bereichs- bzw. länderspezifische Regelungen. Die im Klinikalltag erhobenen Gesundheitsdaten werden als besondere personenbezogene Daten im Sinne des Art. 9 Abs. 1 DSGVO bezeichnet, deren Verarbeitung nur beim Vorliegen einer Rechtsgrundlage (z. B. Einwilligung, Gesetz) zulässig ist. Neben dem Datenschutz ist bei diesen sensiblen Daten auch das Berufsgeheimnis relevant. Ärzte und medizinisches Personal unterliegen nach § 203 Strafgesetzbuch [20] einer besonderen beruflichen Verschwiegenheitsverpflichtung. Ein Verstoß kann strafrechtlich geahndet werden. Die Einhaltung von Datenschutz und Schweigepflicht hat daher sowohl für die betroffenen Patienten als auch für die Beschäftigten im Krankenhaus eine große Bedeutung.

Ziel dieser Arbeit ist es, einen kompakten Überblick darüber zu geben, wie Themen der Informationssicherheit und des Datenschutzes in einen effizienten Klinikalltag integriert werden können. Es soll gezeigt werden, dass das kein Widerspruch ist, jedoch einiger Vorplanung bedarf, bevor es reibungslos funktioniert.

## Methode

Es wird eine narrative Kurzübersicht gegeben.

## Eckpunkte

### Was genau ist Informationssicherheit und was verbirgt sich hinter einem ISMS?

Informationssicherheit bezeichnet den Schutz von Informationen jeglicher Art und Herkunft [9, 10].

Konträr zum Datenschutz geht es nicht nur um den Schutz personenbezogener Daten, sondern um den Schutz aller internen und vertraulichen Daten und Informationen des eigenen Unternehmens. Dazu gehören neben Patientendaten beispielsweise auch Forschungsdaten und Konfigurationsdaten von Medizingeräten [11].

Die Informationssicherheit dient der Sicherstellung der Grundziele Vertraulichkeit, Integrität, Verfügbarkeit und Authentizität von Informationen. Was das konkret im Klinikalltag bedeutet, zeigt Tab. 1.

**Table 1:** 

Schutzziel	Erläuterung	Praxisbeispiele
Vertraulichkeit	Daten dürfen nur von berechtigten Personen eingesehen werden	Persönlicher Login am PC, KIS, Anwendung Verschlüsselung der Daten bei Transport und Speicherung (z. B. Cloudsysteme)
Integrität	Daten dürfen nicht unerkannt bzw. unbemerkt verändert werden	Zugriffsprotokollierung KIS-System
Verfügbarkeit	Daten oder Systeme sind zu dem Zeitpunkt nutzbar, wenn es notwendig ist	„Back-up“ von KIS-Daten IT-Systeme und Medizingeräte stehen immer funktionsfähig zur Verfügung
Authentizität	Daten müssen echt und vertrauenswürdig sein	Personenbezogene Anmeldung an Systemen

Z. B. Computerarbeit, Arzt-Patienten-Gespräch, ärztliche/medizinische Schweigepflicht, Visite, Datenmonitoring, (sporadische, stichprobenartige) Prüfung von Datenvalidität/-güte/-kongruenz, Sichtschutz am Stationstresen etc.

Die Erfüllung dieser Ziele ist eine wichtige Voraussetzung zum Erreichen der für eine Klinik essenziellen Ziele [6]:

- Patientensicherheit (und)
- Behandlungseffektivität.

**Figure 1:**
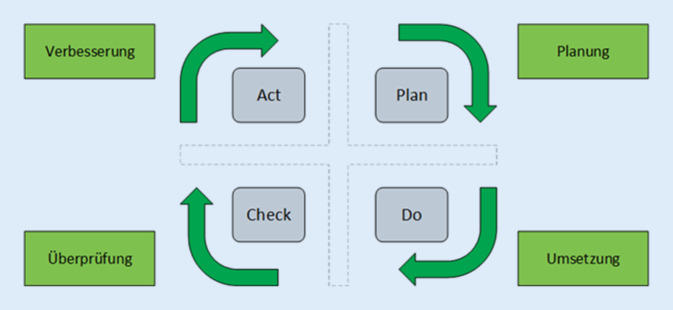

Informationssicherheit ist – zusammenfassend gesagt – also keine einzelne Technologie. Es ist vielmehr eine Strategie, die aus Prozessen, Tools sowie Leit- und Richtlinien besteht [12–14]. Und genau diese Teile der Strategie bilden ein Managementsystem der Informationssicherheit (kurz: ISMS) ab. Dabei unterliegt das ISMS einem ständigen Verbesserungsprozess (Abb. 1). Dafür sind interne und externe Überprüfungen mittels Audits essenziell.

Informationssicherheit funktioniert nur unter der Voraussetzung, und das ist Eigenschaft aller Sicherheitskategorien, dass es klare Regeln und Prozesse gibt, die alle Beteiligten einhalten.

Die Etablierung von Informationssicherheit, und das gilt auch hier wieder für Sicherheitsmechanismen im Allgemeinen, wird gerade im Klinikkontext oftmals als beschränkend erlebt.

Getroffene Regeln und geänderte Prozesse dienen aber niemals dazu, die klinische Arbeit zu erschweren, sondern sie effektiv zu schützen und damit langfristig die gemeinsamen Ziele der Patientensicherheit und die Behandlungseffektivität zu gewährleisten [11].

Ein Vergleich der Arbeit eines Chirurgen von vor 150 Jahren mit der Arbeit in einem modernen Operationssaal macht das deutlich. Aus heutiger Sicht wirken insbesondere die damaligen Hygienemaßnahmen zum Schutz des Operateurs als auch des Patienten geradezu fahrlässig. Allerdings war man sich damals der zahlreichen Bedrohungen einfach nicht bewusst bzw. es fehlten Erkenntnisse und Erfahrungen. Umgekehrt würde ein Chirurg aus der damaligen Zeit die Hygienemaßnahmen und das Operieren mit Schutzkleidung, wie Sie heute selbstverständlich sind, sicher auch als sehr beschwerlich und einschränkend empfinden.

An diesem Beispiel lässt sich leicht die Analogie zur Informationssicherheit herstellen. Wir wissen heute von den verschiedenen Bedrohungsszenarien durch Cyberangriffe und Spionage auf die kritische Infrastruktur Krankenhaus. Demnach müssen wir darauf mit angemessenen Schutzmaßnahmen reagieren [4, 11].

### Wie lässt sich Informationssicherheit also in den Klinikalltag integrieren?

Ein effizienter Klinikalltag und die Integration der Informationssicherheit sind jedoch kein Widerspruch, bedürfen aber einige Vorplanung, bevor es reibungslos funktioniert.

Zunächst ist es wichtig, den Dialog mit dem Informationssicherheitsbeauftragten (ISB) zu suchen. Er muss die klinischen Prozesse verstehen, um Maßnahmen zur Wahrung der Ziele der Informationssicherheit effizient in den klinischen Alltag an spezifischen institutionellen und Kliniklokalisationen integrieren zu können. Dabei dürfen die üblichen Abläufe nicht verlangsamt oder gestört werden.

Dazu kann der ISB zu Hospitationen in den eigenen Bereich eingeladen werden und es können gemeinsam sog. Risikointerviews durchgeführt werden [16]. Hier kann ausgeführt werden, welche Systeme und Applikationen als kritisch, also als unabdingbar für eine optimale Patientenversorgung erachtet werden. Nur so kann der ISB einschätzen, wo welche Maßnahmen getroffen werden müssen und wo diese möglicherweise ganz entfallen können.

Im Rahmen der Hospitationen und der Interviews ist es auch von Relevanz, mögliche vorhandene IT-Probleme darzulegen. Das Lösen dieser Probleme ist die Basis für funktionierende Informationssicherheitsmaßnahmen. Oftmals lassen sich davon auch die Notwendigkeit gezielter Investitionen und der Optimierung der IT ableiten.

Im Ergebnis wird es dadurch gelingen, Maßnahmen in den klinischen Bereichen verhältnismäßig umzusetzen.

### IT-Notfallmanagement – ein Thema für den Chirurgen?

IT-Notfallmanagement bedeutet, dass man im IT-Notfall (ausgelöst z. B. durch andauernden Systemausfall oder Cyberangriff) geordnet und möglichst unterbrechungsfrei in einen vorher definierten klinischen Notfallbetrieb wechselt [5–8].

Auch wenn der Name es vielleicht vermuten lässt, so ist das IT-Notfallmanagement kein Thema, für das nur die IT verantwortlich ist.

**Figure 2:**
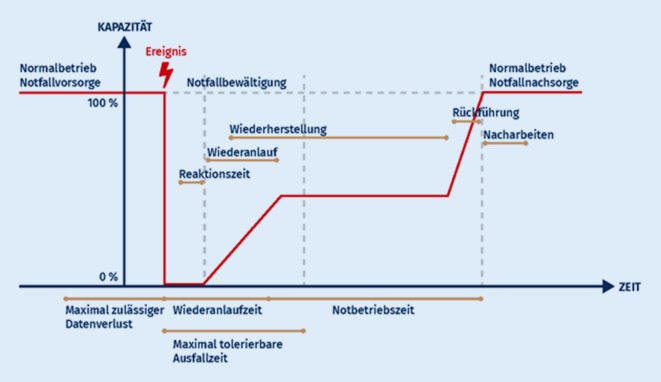

Im Notfallbetrieb werden aus digitalen plötzlich analoge Prozesse und fallen damit nicht mehr in die Verantwortung der IT, sondern der Klinik selbst (Abb. 2).

Jeder Mitarbeiter einer Klinik muss aber die Handlungsabläufe und Prozesse während des IT-Notfallbetriebs kennen, damit die Patientenversorgung und Behandlungseffektivität auch ohne oder nur mit eingeschränkter IT sichergestellt werden kann.

Dazu müssen regelmäßige Übungen stattfinden und ein digitales und analoges IT-Notfallhandbuch in der Klinik verfügbar sein. Für die Koordination der Maßnahmen im IT-Notfall muss zudem die Rolle des IT-Notfallverantwortlichen besetzt werden.

### Was genau ist Datenschutz?

**Figure 3:**
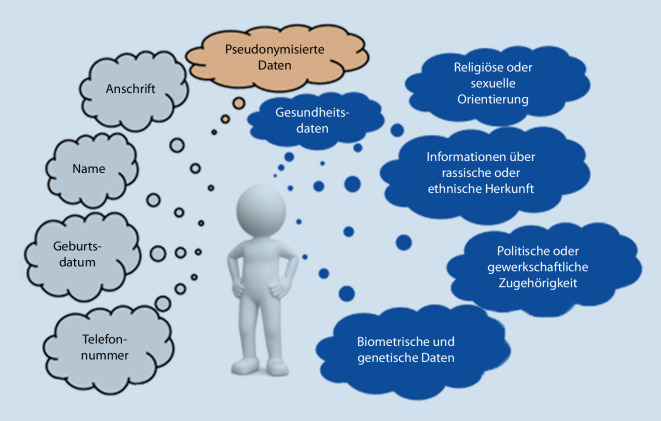

Datenschutz ist ein Grundrecht zum Schutz vor missbräuchlicher Verarbeitung personenbezogener Daten. Dabei ist nicht der Schutz der Daten, sondern das Recht des Einzelnen auf informationelle Selbstbestimmung das Schutzziel. Jeder soll selbst bestimmen können, wem er welche Daten wann und zu welchem Zweck zur Kenntnis gelangen lassen will. Datenschutz ist relevant, wenn personenbezogene Daten im Sinne der DSGVO verarbeitet werden (Abb. 3).

**Figure 4:**
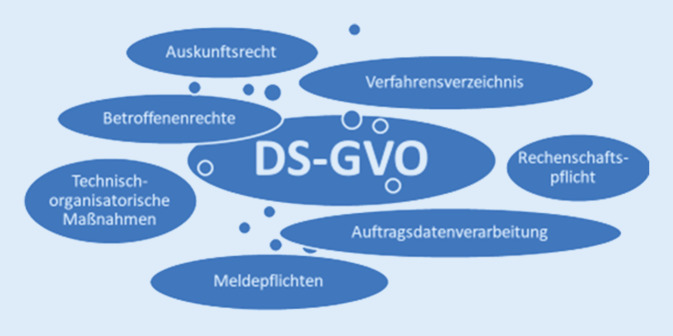

Vorgaben für eine rechtmäßige Datenverarbeitung machen in Deutschland die DSGVO sowie das Bundesdatenschutzgesetz und die Landesdatenschutzgesetze. Inhalt dieser Datenschutzregelungen sind Grundsätze des Datenschutzes, die sowohl die Rechte der Betroffenen als auch technisch-organisatorische Maßnahmen zum Schutz der Daten betreffen (Abb. 4).

### Wie lässt sich Datenschutz in den Klinikalltag integrieren?

Im Klinikalltag werden in zahlreichen Konstellationen Patientendaten erhoben, verarbeitet und/oder an Dritte (z. B. Hausärzte, Krankenversicherung, Behörden, Angehörige) übermittelt. In jedem dieser Fälle muss geprüft werden, ob und unter welchen Bedingungen dies zulässig ist.

Die Rechenschaftspflicht des Art. 5 Abs. 2 DSGVO verlangt, dass der Verantwortliche nachweisen kann, dass er die Datenschutzvorschriften in vollem Umfang befolgt. Voraussetzung für diese Nachweisführung ist die Darlegung, in welchen klinischen Prozessen überhaupt personenbezogene Daten verarbeitet werden.

Was dies konkret im Klinikalltag bedeutet, zeigt Tab. 2.

**Table 2:** 

Klinischer Prozess	Datenverarbeitung	Rechtsgrundlage	Darauf ist achten
*Patientenaufnahme*			
Administrative Aufnahme (geplante Aufnahme, Akut‑/Notfallaufnahme)	– Datenerfassung – Nutzung der Daten von Voraufenthalten – Klärung der Übermittlung der Behandlungsdaten/-informationen an – Vor‑/Nach‑/Weiterbehandler – Angehörige – Seelsorger	– Behandlungsvertrag – Einwilligungen	– Datenerhebung nach Zweckmäßigkeit/Erfordernis – Unterschrift des Patienten auf dem Behandlungsvertrag – Aushändigung einer Kopie des Behandlungsvertrages
Medizinische Aufnahme	– Anamneseerhebung – Status praesens – Aufklärung über vorläufige Diagnose und geplante Therapie – Anordnung verschiedener diagnostischer/therapeutischer Maßnahmen	– Einwilligung § 630d BGB	– Umfang der Datenerhebung liegt im Ermessen des Arztes, sollte Grundsätzen der DSGVO entsprechen
*Gesundheitsversorgende Abläufe/Behandlung*			
Klinik	– Interner Zugriff – Dokumentation von Diagnostik und Therapie – Fallbegleitende Kodierung	– Einwilligung § 630d BGB – § 630f	– Regelung für Zugriffsberechtigungen (Personal) – Keine Offenbarung an sensiblen Patientendaten an Unbefugte – Schutz vor unbefugten Zutritt und Zugriff auf Patientenakten – Beim Verlassen des Arbeitsplatzes PC sperren
Station	– Aufbewahrung der Patientenakte – Interner Zugriff – Dokumentation von Diagnostik und Therapie – Eigene Einsichtmaßnahme – Mehrbettzimmer	– § 630f BGB – Verjährungsvorschriften	– Regelung für Zugriffsberechtigung (Personal) – Patientenakten und Monitore nicht einsehbar an Stationstresen lagern/anbringen – Aktenwagen verschlossen halten – Beim Verlassen des Arbeitsplatzes PC sperren
Auskunft	– Telefonische Auskunft – Krankenhausseelsorger – Angehörige	– Einwilligung	– Auskunft nur nach Legitimation
Entlassung	– Führen/Dokumentation des ärztlichen Entlassungsgesprächs – Erstellung eines Arztbriefes (ggf. Operationsbericht) – Entlassungsmanagement – Abschließende Kodierung – Abrechnung – Übermittlung von Patientendaten an Dritte (Vor‑/Weiter‑/Nachbehandler/KK)	– § 39 Absatz 1a SGB V – § 301 SGB V – § 275 SGB V – Einwilligungserklärung	– Prüfen, ob Einwilligungserklärung zur Übermittlung an Vor‑/Nach‑/Weiterbehandler vorliegt – Datenschutzgerechter Versand der Unterlagen

Z. B.: E‑Mail-Korrespondenz, Dienstplan, Urlaubsplan, Operationsplan, Krankenakten, Arztbriefe, medizinische Befunde, Computerarbeit/-dokumentation im geschützten Umfeld, bestmögliche Vermeidung von Aktensichtbarkeit/-zugriff, Patienten- und pseudonymisierte Daten im Rahmen wissenschaftlicher Studien etc.

Bei jeder Verarbeitung personenbezogener Daten sind die Datenschutzgrundsätze nach Art. 5 Abs. 1 DSGVO einzuhalten. Hierbei handelt es sich um

- Rechtmäßigkeit,
- Verarbeitung nach Treu und Glauben,
- Transparenz,
- Zweckbindung,
- Datenminimierung,
- Richtigkeit,
- Nachvollziehbarkeit, Revisionsfähigkeit,
- Speicherbegrenzung,
- Integrität und Vertraulichkeit,
- Rechenschaftspflicht.

### Welche Folgen kann eine Missachtung datenschutzrechtlicher Vorgaben haben?

Ein Verstoß gegen datenschutzrechtliche Vorgaben kann einschneidende Folgen haben. Die maßgeblichen Bestimmungen für die Sanktionierung von Datenschutzverstößen enthält die DSGVO in Art. 77 ff. Dort ist festgelegt, dass Datenschutzverstöße mit hohen Bußgeldern von bis zu 20 Mio. € oder 4 % des weltweiten Jahresumsatzes der verantwortlichen Stelle geahndet werden können. Nach § 42 BDSG kann sogar eine Freiheitsstrafe von bis zu 3 Jahren verhängt werden. Neben diesen gesetzlichen Sanktionen können auch Ansprüche auf Schadensersatz durch die geschädigten Personen gestellt werden.

Beispiele für verhängte Bußgelder gegen Kliniken zeigt Tab. 3.

**Table 3:** 

Klinik	Verstoß	Bußgeld
Universitätsklinikum Mainz	Verwechslung von Patientendaten bei der Aufnahme, Ausstellen falscher Rechnungen	105.000 €
Barreiro Montiijo, Lissabon	Unzureichende Zugriffsregelungen	400.000 €
Haga Ziekenhaus, Den Haag	Unzureichende technisch-organisatorische Maßnahmen	460.000 €
Klinik, Baden-Württemberg	Abrufbarkeit von Patientendaten im Internet	80.000 €
Capio St. Göran, Schweden	Fehlende Bedarfs- und Risikoanalyse für den Zugriff auf Patientendaten, zu weitreichende Zugriffsberechtigungen	2.895.054 €
Ostfold HF, Norwegen	Zugriffsmöglichkeit auf Patientenakten für alle Mitarbeiter	750.000 €

Verantwortlich für die Einhaltung des Datenschutzes im Klinikalltag sind sowohl die Klinikleitung als auch jeder einzelne Mitarbeiter. Die Klinikleitung trägt die Gesamtverantwortung, insbesondere die Umsetzung technischer und organisatorischer Maßnahmen. Dabei wird sie vom Datenschutzbeauftragen der Klinik unterstützt, der für die Erfüllung seiner gesetzlich vorgeschriebenen Aufgaben verantwortlich ist. Daneben trägt aber auch jeder einzelne Mitarbeiter für die Wahrung des Datengeheimnisses bezüglich der Daten in seinem Bereich Verantwortung.

### Informationssicherheit und Datenschutz als Element des QM

Obwohl Informationssicherheit und Datenschutz keine klassischen Elemente des Qualitätsmanagements (QM) wie

- Struktur‑, Prozess- und Ergebnisqualität,
- Mitarbeiter‑/Kunden-(Patienten‑)Zufriedenheit,
- Fehlermanagement,
- Reorganisationsbestrebungen,
- Beauftragtenwesen (u. a. m.)

sind, finden sie sich in heutige QM-Betrachtungen integriert, da sie im digitalen Zeitalter und nach den Erfordernissen einer modernen Arbeitsorganisation sowie sicherer Arbeitsprozesse/-abläufe unentbehrlich sind. So sind neben den etablierten QM-Audits und -Zertifizierungen auch solche mit Fokus auf Informationssicherheit und Datenschutz sinnvoll und sollten implementiert sowie regelmäßig durchgeführt werden.

### Rolle von Audits

Audits stellen auch in den Bereichen der Informationssicherheit und des Datenschutzes ein geeignetes Instrument dar, um nach den Vorgaben gesetzlicher oder betrieblicher Regelungen

- Mängel zu eruieren,
- (weitere) Vorgaben abzuleiten,
- Erfüllungsstände zu überprüfen (und)
- periodisch den Organisationsstand bezüglich bereits erreichter bzw. etablierter Standards zu prüfen.

### Funktionen und Verantwortlichkeiten im Zusammenhang mit Informationssicherheit und Datenschutz

Bei der Umsetzung von Informationssicherheit und Datenschutz im klinischen Alltag ergibt sich auch für das medizinische Personal Arbeits- und Organisationsaufwand, der personell unterstützt werden müsste.

### Datenschutz- und Informationssicherheitskoordinator der Klinik – eine Funktions- und Tätigkeitsbeschreibung

Um die vielfältigen Themen des Datenschutzes und der Informationssicherheit verhältnismäßig und praxisnah umsetzen und regelmäßig verbessern zu können, ist der Einsatz sog. Datenschutz- und Informationssicherheitskoordinatoren in der Klinik unabdingbar.

Da der Datenschutz gewissermaßen eine Teilmenge der Informationssicherheit ist und um personelle Ressourcen zu schonen, können beide Rollen unproblematisch in einer Person vereint werden. Um dieser Rolle gerecht werden zu können, müssen die Datenschutz- und Informationssicherheitskoordinatoren entsprechend geschult werden.

Welche Aufgaben ein Datenschutz- und Informationssicherheitskoordinator übernehmen soll, verdeutlicht Tab. 4.

**Table 4:** 

Aufgabe	Datenschutz	Informationssicherheit
Informationseinholung im Bereich	☑	☑
Ansprechpartner/Bindeglied	☑	☑
Erhebung bereichsspezifischer Verfahren	z. B. Verzeichnis von Verarbeitungstätigkeiten	z. B. IT-Anwendungen
Umsetzung von Standardprozessen	z. B. Formulare	z. B. Formulare
Aufbereitung von Fragen	☑	☑
Vorfallsaufklärung	☑	☑
Unterstützung Risikobewertung	Datenschutzfolgeabschätzung	Bewertung von Informationssicherheitsrisiken
Unterstützung interner Audits	☑	☑
Unterstützung Schulungen/Awareness	☑	☑
regelmäßige Berichte an …	Datenschutzbeauftragte(n)	Informationssicherheitsbeauftragte(n)

### Ausblick – Trends

Informationssicherheit und Datenschutz werden stets verbindlicher Bestandteil einer adäquaten Arbeitsorganisation bleiben und ihre sehr ernstzunehmende Rolle beibehalten. Nicht zuletzt sind sie stets weiter als relevantes Element im QM anzusehen.

Als sensibilisierende Anregung wird das Beispiel eines „Feldversuchs“ zu einer Supervision eines chirurgischen Arbeits(all)tages unter Sensibilisierung auf Datenschutz und Informationssicherheit vorgestellt (Tab. 5).

**Table 5:** 

Aspekt des klinischen ärztlich-chirurgischen Tagesablaufs	Verstöße/Mängel			Praktische Tipps, Handlungsanweisungen
	Datenschutz	Info-Sicherheit	Fehlerranking	
Fahrstuhlbenutzung zur Station unter Gespräch über kritischen Patienten mit einem Kollegen	+	–	Hoch	Unbedingte Vermeidung von Gesprächen zu dienstlichen oder Patientenbelangen in der Öffentlichkeit unter „Mithöroption“
Visite unter Zimmerwechsel nahe des Stationstresens, wo zwei stationär aufzunehmende Patienten mit ihren Angehörigen warten	+	–	Hoch	Beachtung von Öffentlichkeits„verkehr“ im Stationsablauf
Kurze computerbasierte Anmeldung nach Visitenende bis zum Beginn der morgendlichen Dienstbesprechung mit „bedenkenlosem“ Verlassen des Computerarbeitsplatzes	+	–	Hoch	Computerarbeitsplatz nicht eingeloggt verlassen, um unberechtigte Benutzung nicht dienstrelevanter Funktionen/Websites zu verhindern als auch keinen unberechtigten Zugriff zu sensiblen Patientendaten zu ermöglichen → konsequentes Ausloggen beim Verlassen des Computerarbeitsplatzes
Umziehen in Mitarbeiterschleuse des Operationssaales mit Verbleiben von zwei Patientenbefunddokumenten/Epikrisenausdrucken im Arztkittel	–	+	Mittelhoch	Schriftliche Befunde nicht unbeaufsichtigt „hinterlassen“
Arzt – Schwester-Gespräch in der Mittagspause über kranken Vater einer auf Station arbeitenden Schwester auf der ITS – Nachsehen im einrichtungsinternen Internet wird verworfen	+	–	Hoch	Nicht arbeitsbedingte Patienten(befund)recherche unter Nutzung arbeitsbedingt notwendiger Einloggoptionen aus Patientenschutzgründen nicht zulässig
Laufende Operationsaufklärung durch Assistenzarzt im Patientenzimmer – zu dieser Szenerie tritt die zuständige Zimmerschwester mit einem neu aufzunehmenden Patienten und dessen Angehörigen	+	+	Hoch	Vertrauliche Patientengespräche sind in ruhiger „geschützter“ Atmosphäre zu führen, möglichst störungsfrei(-arm) ohne Option des Mithörens unberechtigter Personen (Angehöriger)
*(Nach‑)Mittags‑/Tageszweitvisite ca. 14:30*				
Eintreten der ärztlich-pflegerischen Visitengruppe ins Patientenzweibettzimmer, wo bei einem der beiden Patienten Angehörigenbesuch weilt	+	–	Mittelhoch	Zur Visitierung des nichtbesuchten Patienten muss der besuchende Angehörige vor die Tür gebeten werden
				Für die Visite beim besuchten Patienten muss dieser Patient befragt werden, ob das Verweilen des Besuches akzeptiert wird (keinesfalls für sensible Untersuchungsakte unter Entblößen, Wundkontrolle/Verbandswechsel etc.)
Beim Zimmerwechsel wird der Stationsarzt vor dem nächsten Patienteneinzelzimmer vom Angehörigen des (bei vollem Bewusstsein befindlichen, nicht betreuten) Patienten zur Tumorprognose angesprochen	+	–	Mittelhoch	Ein Patientengespräch „hinterm Rücken des Patienten“ erscheint nicht zulässig (von ganz wenigen Ausnahmen abgesehen: psychische/nichtorientierte Patientensitiuation) – eine Vertrauenslage mit dem Patienten könnte massiv gestört werden, wenn dieser das Angehörigengespräch unter seinem Ausschluss zufällig/unbeabsichtigt mitbekommt

## Fazit

- Informationssicherheit und Datenschutz sind gesetzlich vorgeschriebene und unverzichtbare Elemente allgemeinen, beruflichen und sozialen Handelns, die auch ins QM eingehen, denen auch der (Allgemein‑/Viszeral‑)Chirurg in seinem Wirken untersteht:
  Die* Informationssicherheit* bezeichnet den Schutz von Informationen jeglicher Art und Herkunft – sie dient der Sicherstellung der Grundziele Vertraulichkeit, Integrität, Verfügbarkeit und Authentizität von Informationen (z. B.: Computerarbeit, Arzt-Patienten-Gespräch, ärztliche/medizinische Schweigepflicht, Visite, Datenmonitoring, [sporadische, stichprobenartige] Prüfung von Datenvalidität/-güte/-kongruenz etc.).
  Beim *Datenschutz* handelt es sich um ein Grundrecht zum Schutz vor missbräuchlicher Verarbeitung personenbezogener Daten – dabei ist nicht der Schutz der Daten, sondern das Recht des Einzelnen auf informationelle Selbstbestimmung das Schutzziel (z. B.: E‑Mail-Korrespondenz, Dienstplan, Urlaubsplan, Operationsplan, Krankenakten, Arztbriefe, medizinische Befunde, Patienten- und pseudonymisierte Daten im Rahmen wissenschaftlicher Studien etc.).
  Das* IT-Notfallmanagement* umfasst, dass im IT-Notfall (ausgelöst z. B. durch andauernden Systemausfall oder Cyberangriff) geordnet und möglichst unterbrechungsfrei in einen vorher definierten klinischen Notfallbetrieb gewechselt werden kann.
- Von einem regelmäßigen bilateralen Austausch zwischen dem Chirurgen und den Beauftragten für Informationssicherheit und Datenschutz profitieren beide Seiten gleichermaßen.
- Durch eine verhältnismäßige Integration von Informationssicherheit und Datenschutz in den klinischen Alltag ist es möglich, die gemeinsamen Ziele der Klinik sowie der Informationssicherheit und des Datenschutzes sicherzustellen:
  Patientensicherheit (und)
  Behandlungseffektivität.
